# Supplementary material for: A Pilot Study on the Feasibility of an Extended Suckling System for Pasture-Based Dairies
Source: Animals (Basel). 2023 Aug 9;13(16):2571. doi: 10.3390/ani13162571 (PMC10451218; doi:10.3390/ani13162571)
Supplement: Supplementary file 1 [file animals-13-02571-s001.zip › animals-2526197-supplementary.pdf]

animals
MDPI

Supplementary material

A Pilot Study on the Feasibility of an Extended Suckling System for Pasture-Based Dairies

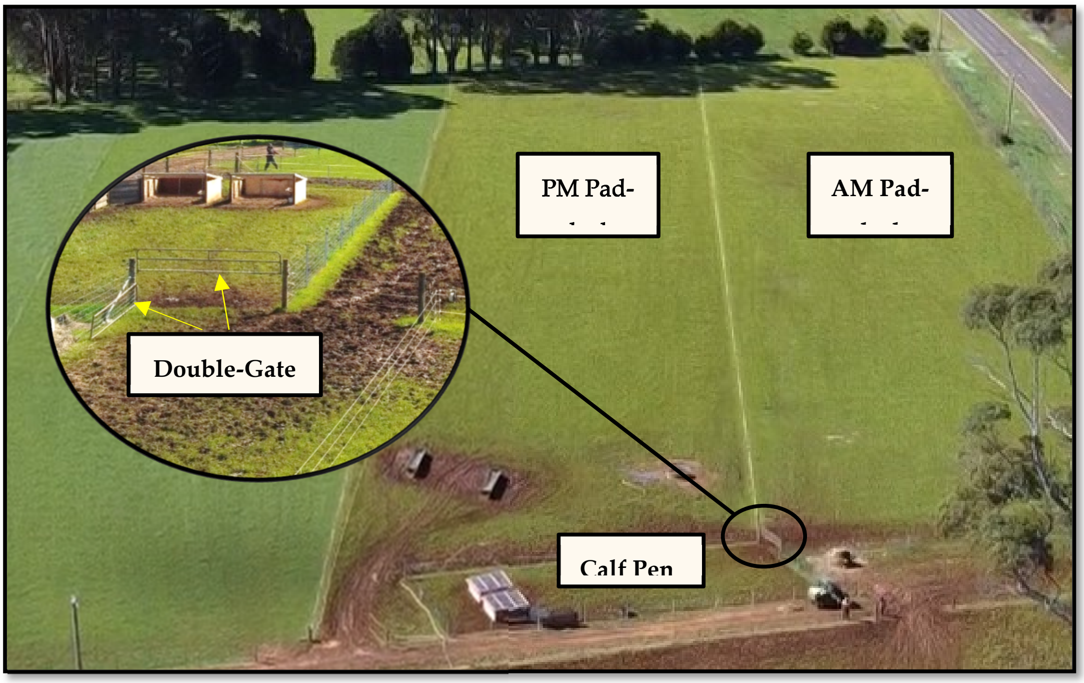

Figure S1. The TIA cow-calf suckling system paddocks used to house the cow-calf pairs.

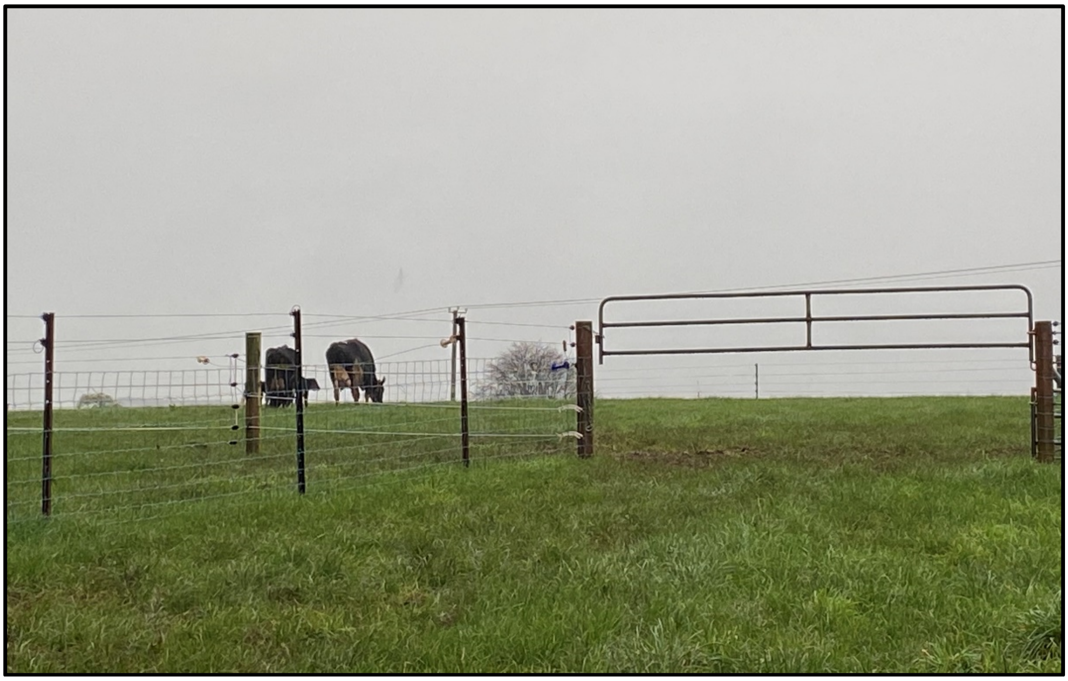

Figure S2. The "double-gate" at opening of the calf pen which prevented cow access to the calf pen while permitting free movement of calves.

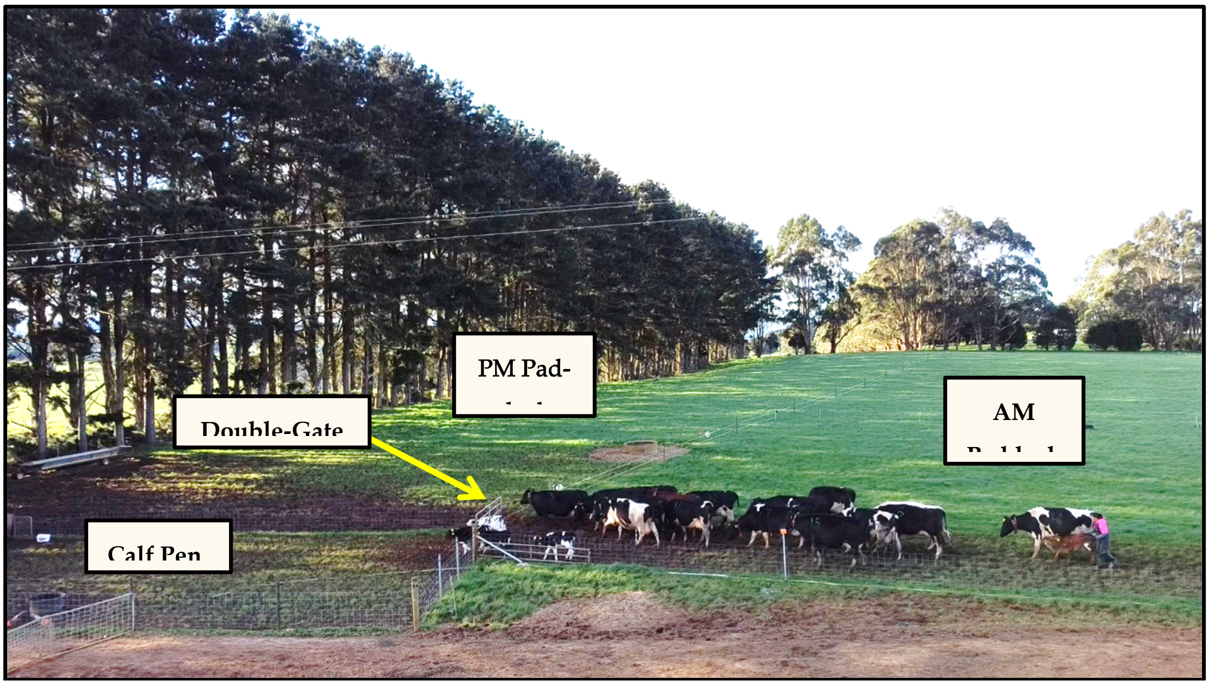

Figure S3. Evening cow-calf separation.

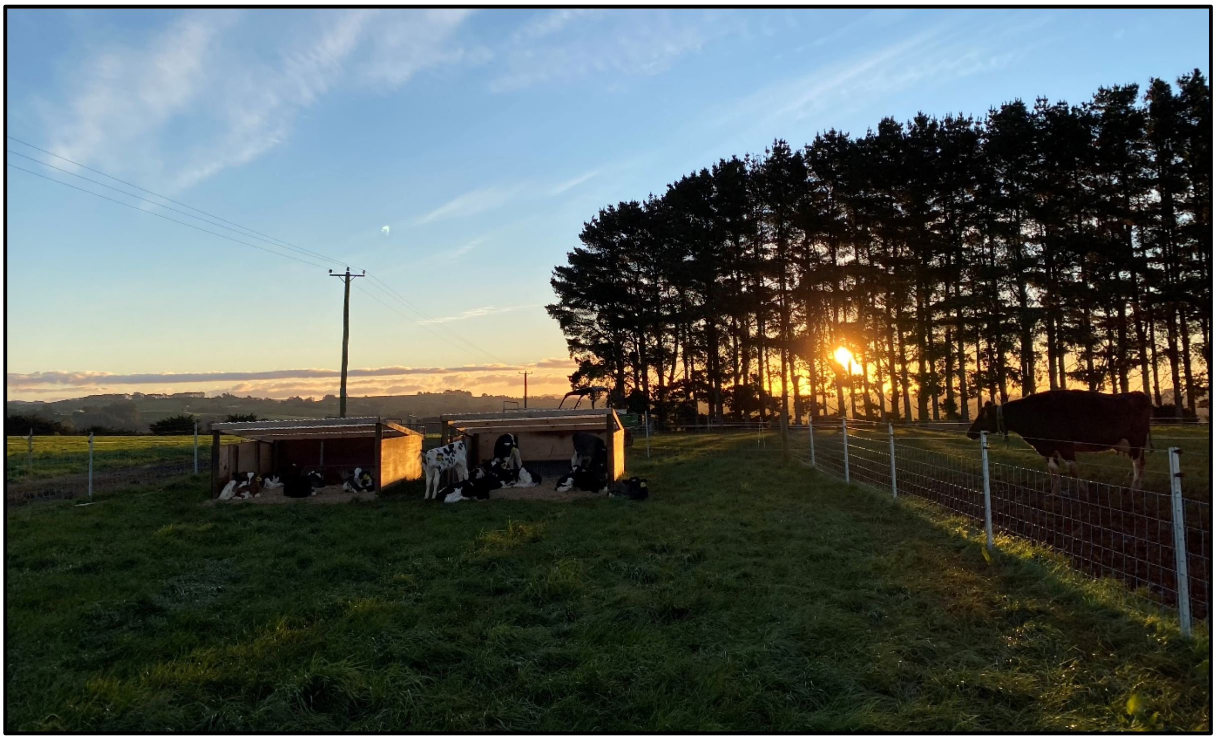

Figure S4. Pasture-based extended suckling system calf pen and overnight fence-line contact.

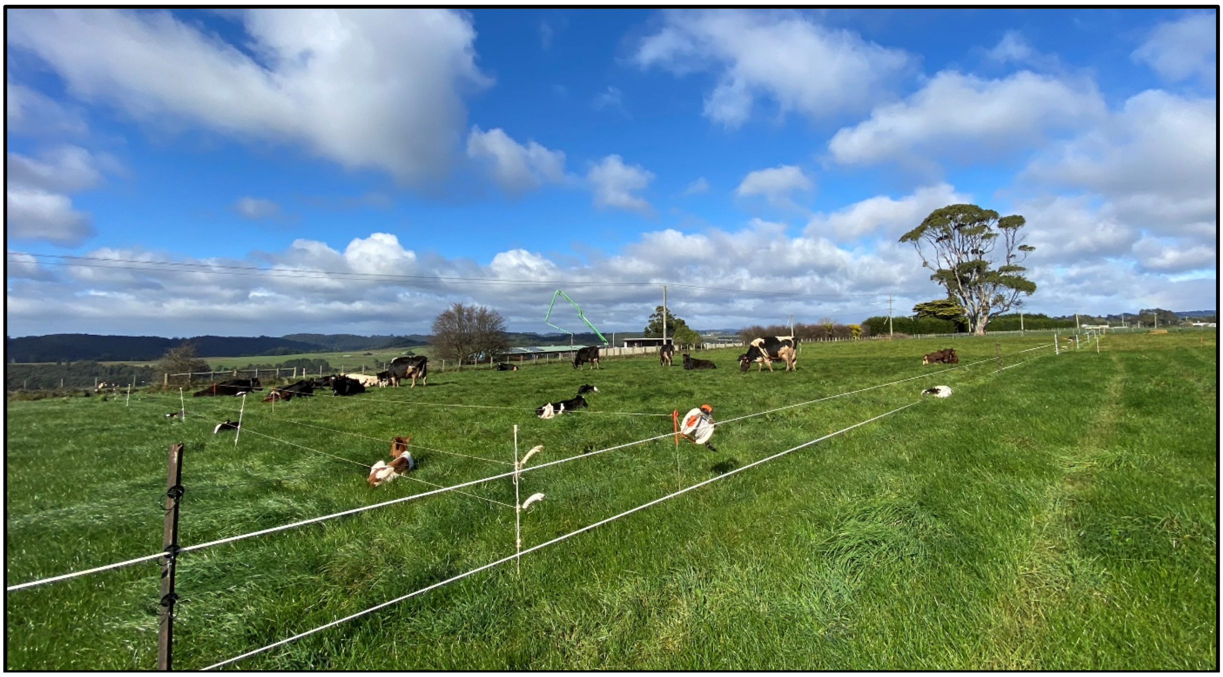

Figure S5. Cow-calf pairs at the AM paddock after milking. Cows and calves spent the day with full contact.

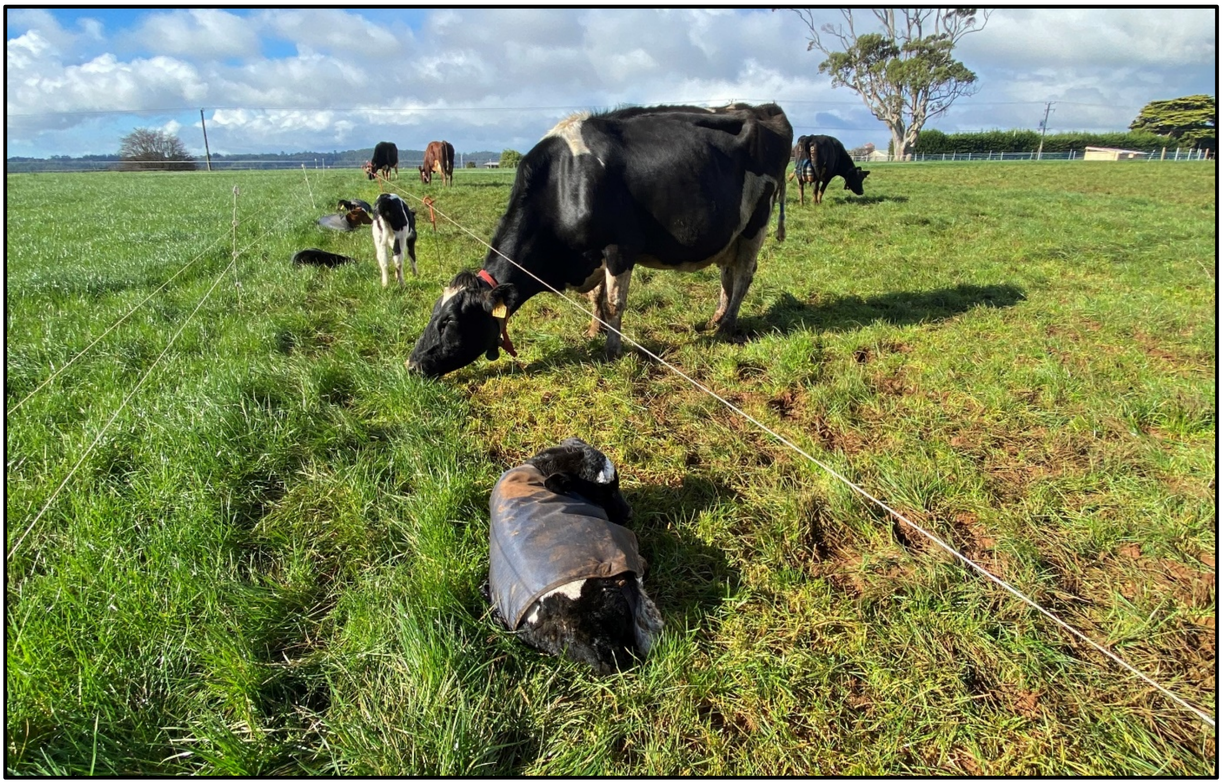

Figure S6. The one-metre strip of tall grass was made accessible only to calves at the front of the AM allocation. Calves were fitted with waterproof jackets during the first 2 weeks of rearing.
